# The prognostic value of red blood cell distribution width in patients with suspected infection in the emergency department

**DOI:** 10.1186/s12873-019-0293-7

**Published:** 2019-12-03

**Authors:** Jan Willem Uffen, Patrick Oomen, Marieke de Regt, Jan Jelrik Oosterheert, Karin Kaasjager

**Affiliations:** 1 Department of Internal Medicine, division Acute Medicine, University Medical Centre Utrecht, Heidelberglaan 100, 3584 CX Utrecht, the Netherlands; 2 Department of Internal Medicine, division Infectious Disease, University Medical Centre Utrecht, Heidelberglaan 100, 3584 CX Utrecht, the Netherlands

**Keywords:** Infection, Sepsis, Emergency department, Emergency medicine, Internal medicine, Biomarkers

## Abstract

**Background:**

Sepsis is a potential life threatening dysregulated immune response to an infection, which can result in multi-organ failure and death. Unfortunately, good prognostic markers are lacking in patients with suspected infection to identify those at risk. Red blood cell distribution width (RDW) is a common and inexpensive hematologic laboratory measurement associated with adverse prognosis in multiple diseases. The aim of this study was to determine the prognostic value of RDW for mortality and early clinical deterioration in patients with a suspected infection in the emergency department.

**Methods:**

In this single center prospective observational cohort study, consecutive patients with suspected infection presenting for internal medicine in the emergency department between September 2016 and March 2018 were included. For prognostic validation of bedside sepsis scores and RDW receiver operating characteristics were generated. Association between RDW and mortality and ICU admission was analyzed univariate and in a multivariate logistic regression model.

**Results:**

1046 patients were included. In multivariate analyses, RDW was significantly associated with 30-day mortality (OR 1.15, 95% CI: 1.04–1.28) and early clinical deterioration (OR 1.09, 95% CI: 1.00–1.18). For 30-day mortality RDW had an AUROC of 0.66 (95% CI 0.59–0.72). Optimal cut-off value for RDW 2 was 12.95%. For early clinical deterioration RDW had an AUROC of 0.59 (95% CI 0.54–0.63) with an optimal cut-off value of 14.48%.

**Conclusions:**

RDW was found to be a significant independent prognostic factor of 30-day mortality and early clinical deterioration in patients with suspected infection.. Therefore it can be a used as an extra marker besides bedside sepsis scores in identifying patients at risk for worse outcome in patients with suspected infection.

## Background

Sepsis is a clinical syndrome currently defined as a life-threatening organ dysfunction caused by a dysregulated host immune response to infections [1]. Sepsis has a high incidence, with up to global estimates of 31 million sepsis and 19 million severe sepsis cases, resulting in 5 million deaths annually [2].

Unfortunately, no good diagnostic tool is available for early identification of patients with sepsis and a golden diagnostic standard does not exist [3–6]. In clinical practice, prognostic sepsis scores are often used to identify patients in need of immediate treatment. The Systemic Inflammatory Response Syndrome (SIRS) score, introduced in 1992 and updated in 2001 proved to be insufficiently specific to correctly identify patients most at risk of dying. The prognostic quick Sequential Organ Failure Assessment (qSOFA) score, introduced in 2016 to overcome this problem, lacks sensitivity to identify all patients that are the most at risk of developing sepsis and thus require immediate treatment [1, 7, 8]. The National Early Warning Score (NEWS) or Modified Early Warning Score (MEWS) have been suggested as better performing alternatives in the Emergency Department (ED) [9, 10] and use is adopted by many hospitals but they are not part of standardized care yet. Therefore, there is an urgent need for better prognostic markers in sepsis that can be used at bedside.

Red Blood Cell Distribution Width (RDW), a quantitative measure of variability in size of circulating erythrocytes usually determined in a complete blood count (CBC), is an inexpensive and readily available measurement, that may act as a prognostic factor in several diseases. For example, elevated RDW has been associated with adverse prognosis in various non-infectious [11–17] and infectious diseases [18–20], and in patients diagnosed with (severe) sepsis and septic shock [21–26]. But it has also been demonstrated to be of prognostic value in undetermined populations [27]. The exact pathophysiologic mechanism underlying this association is unclear but systemic factors that alter erythrocyte homeostasis such as inflammation and oxidative stress, both essential components of the infection cascade, seem to play an important role [28–36].

Though the independent association with adverse prognosis in various diseases and sepsis has been established, previous research has focused mainly on well-defined populations such as severe sepsis or septic shock, which hampers the generalizability as a prognostic tool in ED populations. Here, we determine the prognostic value of RDW in patients with suspected infection in the emergency department.

## Methods

### Study design and setting

A retrospective analysis of data from the SPACE-cohort (SePsis in the ACutely ill patients in the Emergency department) between September 2016 and March 2018, was performed. Within the SPACE-cohort, all consecutive patients, age ≥ 18 years, with a suspected infection presenting for internal medicine in the ED of the University Medical Centre Utrecht (UMCU) since September 2016 are prospectively included. The UMCU is a 1042-bed tertiary academic teaching hospital in the Netherlands with approximately 20.500 annual ED visits.

No exclusion criteria were used. Triage for sepsis was performed for all patients presenting for internal medicine in the ED. When sepsis was suspected protocolled care according to a care pathway was initiated. All other patients received protocolled care according to their clinical presentation. The SPACE cohort was reviewed and approved by the Medical Ethical Committee of the UMCU under number 16/594 and registered in the Dutch Trial Register (NTR) under number 6916.

### Population and data collection

The SPACE cohort consists of all consecutive patients who meet the following criteria: (1) ≥ 18 years or older; (2) presentation at the ED with suspected infection defined by the treating physician in the ED; (3) registration in the ED for the internal medicine department or its subspecialties oncology, rheumatology, immunology, hematology, nephrology, endocrinology, gastro-enterology, geriatrics, infectious disease and vascular medicine. All patients received standard care.

The domain of patients with suspicion of infection by their treating physician was deliberately chosen, as this is the exact group a clinician would like to diagnose or rule out the presence of sepsis. Suspicion of infection was documented for all patients by the attending physician at the ED using an automated record system asking this question followed by the question whether a sepsis was present at the time of presentation or not. When both questions are answered the record system automatically adds the parameters needed to calculate SIRS and qSOFA to the patient record. Furthermore the record system gives a warning to the physician when one or both scores are abnormal.

Independent trained physicians analyzed all patients records on documented suspected infection or sepsis. In absence of documentation, the independent physicians registered both items. Suspicion of infection was considered present, when respectively infection or sepsis was recorded by the treating physician in the ED as diagnosis and/or differential diagnosis in the patient record.

For all patients, demographic parameters such as age and gender and clinical parameters such as temperature, heart rate, respiratory rate, saturation, blood pressure, Glasgow coma scale (GCS), laboratory results including RDW, hospital or intensive care unit (ICU) admission and follow-up data on mortality were automatically collected from electronic medical records. SIRS and qSOFA scores were automatically calculated based on the first recorded data.

Data on comorbidities and immunocompromised status were manually extracted from the patient record system by researchers, using a predefined set of well-described definitions. If GCS was not registered, free text notes by the treating physician on the ED were used to derive information on the mental status from.

If data was missing on parameters needed to calculate sepsis scores such as SIRS and qSOFA we chose to score these parameters as normal under the assumption that if patients had abnormal parameters clinicians would have entered these into the electronic medical file.

### Laboratory measurements

Laboratory parameters such as blood chemistry and CBC, including RDW, were performed when patients presented in the ED, and the results were obtained within 1 h. RDW is calculated as the standard deviation of erythrocyte volume divided by the mean corpuscular volume (MCV). The reference range of RDW varies between 10.0–16.0%, depending on the used analyzer. In this study, RDW was measured as part of the automated CBC using the CELL-DYNN Sapphire hematology analyzer (Abbott Diagnostics, Santa Clara, CA, USA). The reference range in the UMCU is 10.5 to 13.5%.

### Outcomes

The primary outcome of this study was defined as all-cause mortality within 30 days after ED presentation. The secondary outcome was defined as early clinical deterioration defined as death or admission to either ICU or Medium Care Unit (MCU) within 3 days after ED presentation.

### Statistical analysis

Continuous data were expressed as mean ± standard deviation (SD) if normally distributed or median and interquartile range in the case of non-parametric data. Independent samples t-tests and Mann-Whitney U tests were used to compare parametric and non-parametric continuous variables, respectively. Categorical variables were compared using Chi-Square or Fisher’s exact test, depending on cell counts.

The association between RDW and outcomes was studied in a multivariate binary logistic regression model, together with variables with a *p*-value of < 0.2 in univariate analysis and clinically relevant variables.

For prognostic validation of SIRS, qSOFA, MEWS and RDW receiver operating characteristics (ROC) curves were generated, and the area under receiver operating characteristic (AUROC) was calculated.

Optimal cut-off value of RDW was determined by using Youden’s J-statistic in above mentioned ROC curve.

All statistical analyses were performed using SPSS version 25.0 (IBM Corp., Armonk, NY). *P*-values < 0.05 were considered statistically significant.

## Results

### Patient characteristics

During the study period, 1119 patients were included in the SPACE cohort, of which 1048 (93.5%) had an RDW measurement at ED presentation and were therefore available for further analysis. In the study cohort 53.8% (*n* = 563) of patients were male and the median age is 61 years old (IQR 50–72). Table 1 shows the descriptive characteristics of the study population, divided in survivors and non-survivors.

**Table 1:** Baseline characteristics of the study population according to survival and non-survival at 30-days

	Total (*n* = 1046)	Non-survivors (*n* = 61)	Survivors (*n* = 985)	*p*-value
Gender (male) – no. (%)	563 (53.8)	37 (60.7)	526 (53.4)	0.270
Age – yr - median (IQR)	61.0 (50–72)	68.0 (61.5–78.0)	61.0 (46.0–69.0)	< 0.001
Comorbidities				
Hematologic malignancy – no. (%)	178(17.0)	10 (16.4)	168 (17.1)	0.960
Chronic renal failure ≥ stage 3 – no. (%)	204(19.5)	13 (22.4)	191 (19.4)	0.713
Immunocompromised – no. (%)	392 (37.5)	15 (24.6)	377 (38.3)	0.092
Neutropenia due to systemic chemotherapy – no. (%)	45 (4.3)	1 (1.7)	44 (4.5)	0.999
CCI - median (IQR)	4.0 (2–7)	7.0 (5–9)	4.0 (2–6)	< 0.001
Vasopressor need – no. (%)	36 (3.4)	8 (13.1)	28 (2.8)	< 0.001
RDW (%) median (IQR)	13.42 (12.30–15.11)	14.74 (13.23–16.71)	13.38 (12.27–14.99)	< 0.001
Disease severity scores				
qSOFA ≥2 – no (%)	82 (7.8)	16 (26.2)	66 (6.7)	< 0.001
SIRS score ≥ 2 – no (%)	656 (58.6)	46 (75.4)	610 (61.9)	0.035
MEWS – median (IQR)	2 (1–4)	3 (1–5)	2 (1–4)	< 0.001
Sepsis according to emergency physician – no. (%)	160 (15.3)	17 (27.9)	143 (14.5)	0.005

*No* number, *Yr* year, *IQR* Interquartile range, *CCI* Charlson Comorbidity Index, *qSOFA* quick Sequential (Sepsis-related) Organ Failure Assessment, *SIRS* Systemic Inflammatory Response Syndrome, *MEWS* Modified Early Warning Score, *RDW* Red Blood cell Distribution Width

Compared to non-survivors, surviving patients are slightly younger (median age 61.0 versus 68.0 (*p* < 0.001)), have a lower Charlson Comorbidity Index (CCI) (median 4.0 versus 7.0, *p* < 0.001) and more often had a lower qSOFA, SIRS score and MEWS at ED presentation (qSOFA ≥2: 6.7% versus 26.2%, *p* < 0.001); (SIRS score ≥ 2: 61.9% versus 75.4%, *p* = 0.035); (MEWS: 2 versus 3, *p* < 0.001). Median RDW at ED presentation was 13.42% (IQR 12.02–14.83). Survivors had a significantly lower RDW (13.38 IQR 12.27–14.99) compared to non-survivors (14.74 IQR 13.23–16.71, *p* < 0.001).

### Primary outcome

**Fig. 1:**
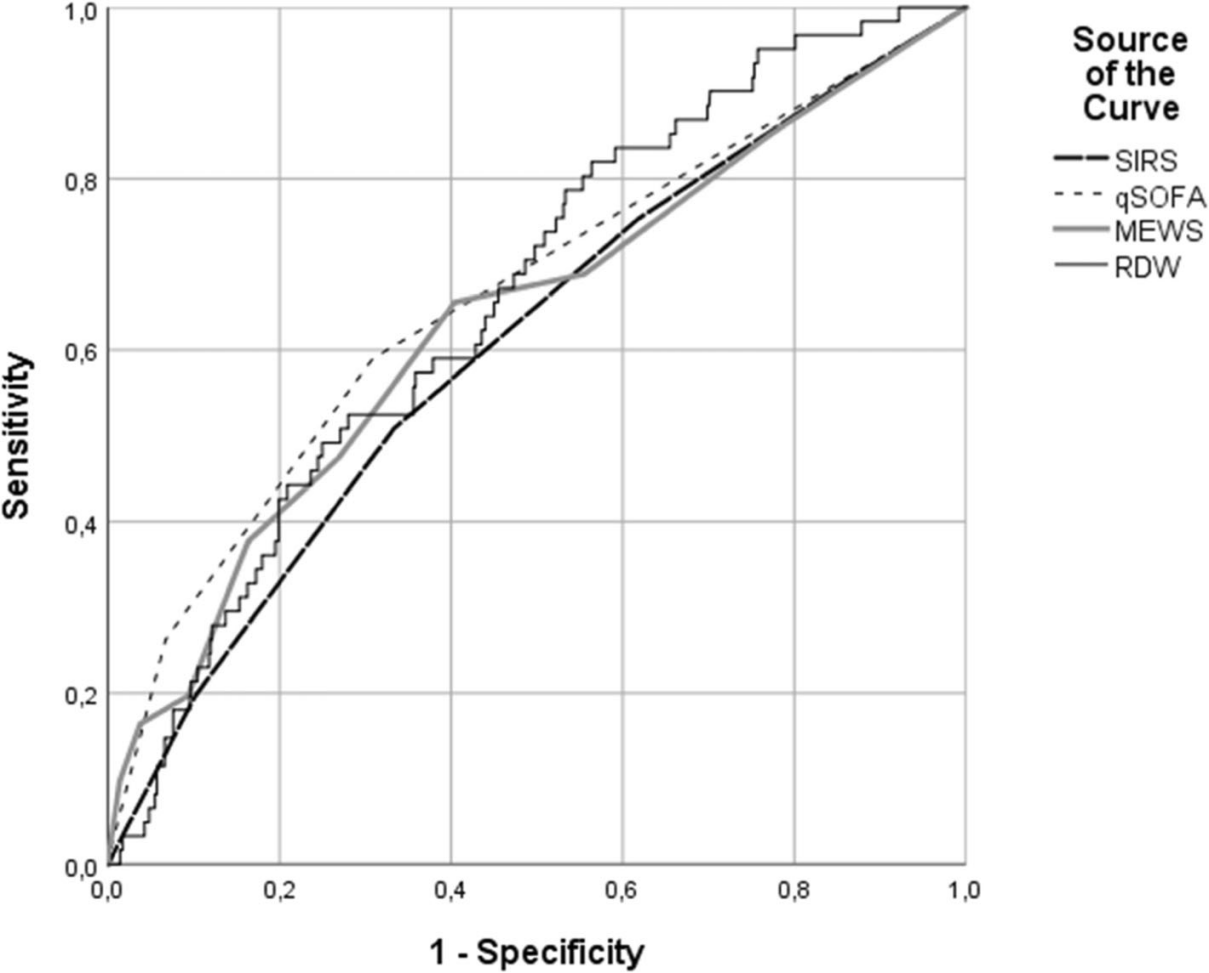
Receiver operator characteristic (ROC) curve for prediction of 30-day mortality. shown are ROC curves of SIRS, qSOFA, MEWS and RDW for 30-day mortality

In total, 61 (5.8%) patients died within 30 days. For 30-day mortality a SIRS score ≥ 2 had a specificity of 0.38 (95% CI 0.35–0.41) and sensitivity of 0.75 (95% CI 0.62–0.85). qSOFA ≥2 had a specificity of 0.95 (95% CI 0.94–0.96) and a sensitivity of 0.26 (0.16–0.39). In an univariate logistic regression analysis age, CCI, vasopressor needs, qSOFA ≥2, a SIRS score ≥ 2, MEWS and RDW were associated with 30-day mortality. Multivariate analysis resulted in age, CCI, vasopressor needs, MEWS and RDW as independent factors associated with 30-day mortality (Table 2). ROC curves for 30-day mortality of RDW, the SIRS score, the qSOFA and the MEWS are shown in Fig. 1 and corresponding AUROCs are shown in Table 3. The optimal cut-off value of RDW was determined at 12.95%.

**Table 2:** Multivariate logistic regression analysis for 30-day mortality

	Odds Ratio	95% CI	*p*-value
Age	1.03	1.00–1.05	0.03
CCI	1.19	1.07–1.32	0.00
Immunocompromised	0.59	0.30–1.12	0.11
Vasopressor needs	2.97	1.12–7.91	0.03
qSOFA ≥2	–		
SIRS score ≥ 2	–		
MEWS	1.22	1.08–1.37	0.00
RDW	1.15	1.04–1.28	0.01

*CCI* Charlson Comorbidity Index, *qSOFA* quick Sequential (Sepsis-related) Organ Failure Assessment, *SIRS* Systemic Inflammatory response syndrome, *MEWS* Modified Early Warning Score, *RDW* Red Blood cell Distribution Width

**Table 3:** AUROCs of RDW, SIRS, qSOFA and MEWS

	Primary outcome		Secondary Outcome	
	AUROC	95% CI	AUROC	95%CI
RDW	0.66	0.59–0.72	0.59	0.54–0.64
SIRS score	0.61	0.53–0.68	0.68	0.63–0.72
qSOFA	0.66	0.59–0.74	0.76	0.71–0.81
MEWS	0.64	0.58–0.71	0.74	0.69–0.78

*RDW* Red cell Distribution Width, *SIRS* Systemic Inflammatory Response Syndrome, *qSOFA* quick Sequential (Sepsis-related) Organ Failure Assessment, *MEWS* Modified Early Warning Score

### Secondary outcome

**Fig. 2:**
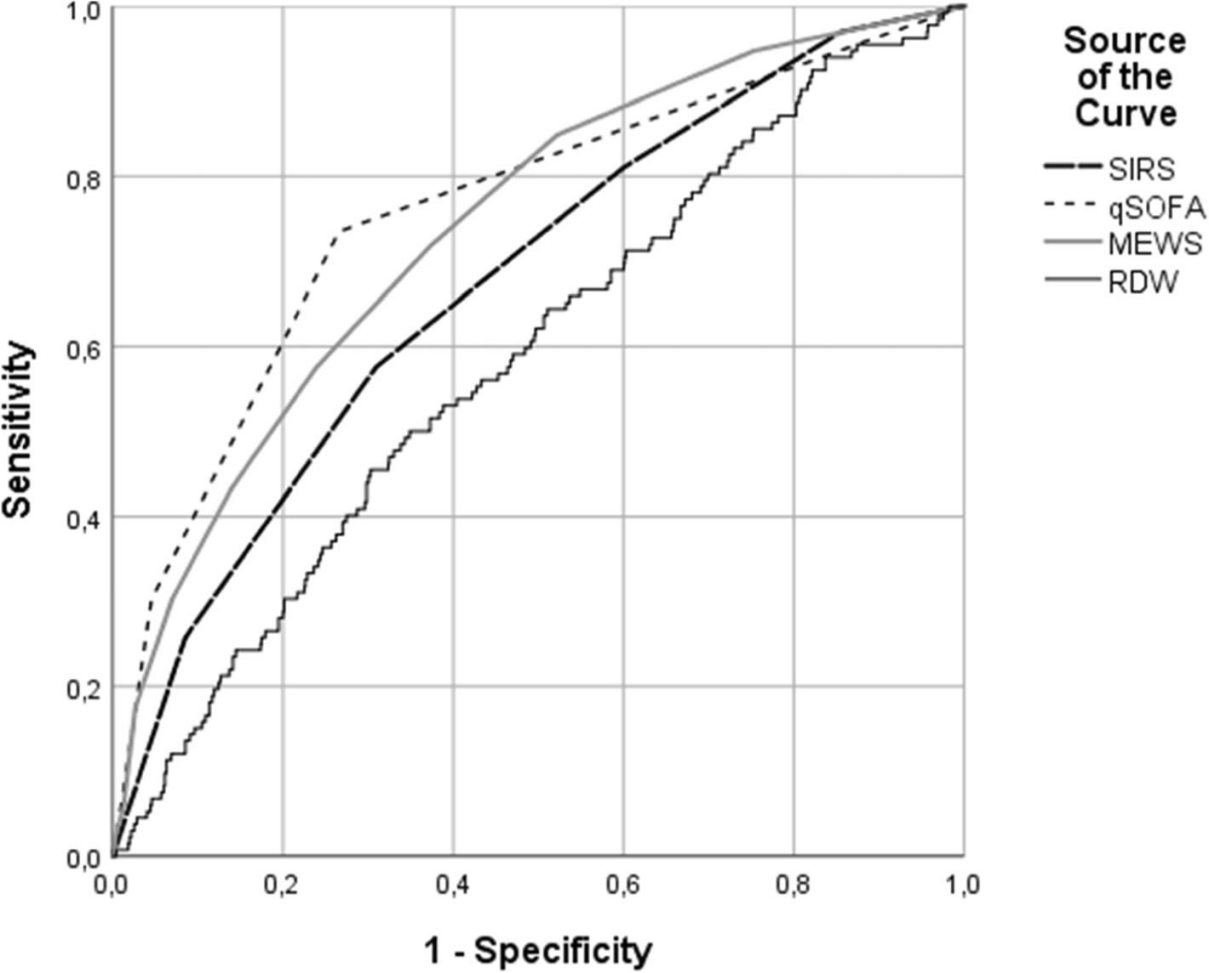
Receiver operator characteristic (ROC) curve for early clinical deterioration. shown are the ROC curves of SIRS, qSOFA, MEWS and RDW for prediction of early clinical deterioration

In this study, 132 (12.6%) patients died within 3 days after ED presentation or were admitted to the ICU/MCU as proxy for early clinical deterioration. Compared to patients with no early deterioration they were older (median age 66 versus 60 years, *p* < 0.001) and more frequently had a SIRS score ≥ 2 (81.1% versus 60.1%, *p* < 0.001), a qSOFA ≥2 (30.3% versus 4.6%, *p* < 0.001) and a higher MEWS (2 versus 4, *p* < 0.001). Furthermore, patients had higher median RDW-levels (14.04% (IQR 12.61–15.92) vs. 13.37% (IQR 12.26–14.98), *p* = 0.001). In a multivariate logistic regression model RDW was associated with early clinical deterioration (OR 1.09 (95% CI 1.00–1.18)). ROC curves for early clinical deterioration of SIRS, qSOFA, MEWS and RDW are shown in Fig. 2 with corresponding AUROCS in Table 3.

Table 4 shows the factors associated with the secondary outcome. Optimal cut-off point for RDW on clinical deterioration was determined at 14.48%.

**Table 4:** Multivariate logistic regression analysis for ICU/MCU admission or death within 3 days

	Odds Ratio	95% CI	*p*-value
Gender (F)			
Age	1.01	1.00–1.03	0.03
Hematologic Malignancy	0.50	0.26–0.96	0.04
Neutropenia due to systemic chemotherapy	0.23	0.04–1.5	0.23
CCI			
Vasopressor needs	16.27	6.70–39.5	0.00
qSOFA ≥2	2.03	3.60–10.11	0.00
SIRS ≥2	–		
MEWS	1.32	1.20–1.46	0.00
RDW (%)	1.09	1.00–1.18	0.06

*CCI* Charlson Comorbidity Index, *qSOFA* quick Sequential (Sepsis-related) Organ Failure Assessment, *SIRS* Systemic Inflammatory response syndrome, *RDW* Red Blood cell Distribution Width

## Discussion

RDW is a common, inexpensive and relatively fast available laboratory measurement and in ED settings in patients with suspected infection it is independently associated with 30-day mortality and early clinical deterioration. Additional in our multivariate analysis the MEWS was also independently associated with 30-day mortality, as SIRS ≥2 and qSOFA ≥2 were not.

The prognostic power of RDW for 30-day mortality is comparable to that of SIRS, qSOFA and MEWS, but for early clinical deterioration RDW is outperformed by all these bedside scores. Our results are in line with previous studies, that showed an independent prognostic value of RDW in sepsis and comparable AUROCs. However, previous studies investigated more defined homogeneous patient groups (e.g. diagnosed sepsis or septic shock) and more severely ill patients. In patients with established diagnose of severe sepsis or septic shock in the ED, non-survivors had significantly higher RDW-levels [23, 25, 26]. In ICU patients with severe sepsis or septic shock, higher RDW-levels were also observed in non-surviving patients [21, 22, 24]. Our study shows that the independent prognostic value also applies to a more heterogeneous group of patients with suspected infection.

Higher sepsis scores are associated with higher RDW-levels suggesting more sick patients have higher RDW-levels. Even though this suggest an association between RDW and higher scores, RDW still remained an independent predictor for mortality after correction for disease severity.

The present study has several strengths. To our knowledge, this is the first study that examines RDW in a heterogeneous population, in which we did not use any exclusion criteria. The study population consisted of patients who were suspected of infection and were admitted to the ED. This is the exact patient population seen daily in EDs and exactly these patients are most at risk of developing sepsis. Therefore results obtained from this study are directly applicable to the daily practice. Additionally, our study had a fairly large sample size which made for precise results. Furthermore, all-cause mortality is an unbiased and relevant outcome in observational prognostic studies.

Certain limitations apply to the current study. Firstly, this was a retrospective single center study based on data from electronic medical records, which is a potential source of bias and errors at the time of data recording at the source. Furthermore, due to the heterogeneous population, there is an increased risk that not all confounding factors have been accounted for. Also, being a single-center study and conducted in a large tertiary care institution, results may not be generalizable to other health care institutions.

Although we took conditions that could have influenced RDW-values into account (i.e. hematologic malignancies, chronic renal failure and neutropenia), RDW-values also could have been influenced by nutritional status. This study did not take iron, vitamin B12, folic acid, erythropoietin levels into account. Additionally, blood transfusion records were not available. Though we did not correct for these factors this study shows that RDW-values are applicable for a heterogeneous group of patients and certain comorbidities or conditions should be taken into account when interpreting RDW-values.

Furthermore, a percentage of qSOFA (2,8%) and SIRS-scores (25,5%) could not be determined due to missing data on respiratory rates. We chose to score these as normal and analyzed the at least achieved score results. This under the assumption that if patients would have been tachypnoeic, clinicians would have entered the respiratory rate into the electronic medical file. Missing data in the ED on vital signs is a well-known problem. Especially completing all vital parameters in one single patient. Failure to register vital signs more often when they appear to be normal. Lower triage categories and less sick patients are associated with lesser recording of vital signs [37]. This could have led to underestimation of SIRS scores and to lesser extent qSOFA. To overcome this problem a complete case analysis has been performed which led to no different results.

## Conclusion

RDW, a common and inexpensive laboratory measurement, usually determined in the CBC, was found to be a significant independent prognostic factor of 30-day mortality and early clinical deterioration in patients with suspected infection and has comparable prognostic accuracy compared to clinical sepsis scores as SIRS qSOFA or MEWS in predicting 30-day mortality. Therefore it can be a used as an extra marker besides these bedside scores in identifying patients at risk for worse outcome.

## Data Availability

The datasets generated and/or analyzed during the present study are not publicly available, but they are available from the corresponding author on reasonable request.

## Abbreviations

AUC: Area under the curve
CBC: Complete blood count
CCI: Charlson comorbidity index
CI: Confidence interval
ED: Emergency department
ICU: Intensive care unit
MCU: Medium care unit
OR: Odds ratio
qSOFA: Quick Sequential Organ Failure Assessment
RDW: Red blood cell differentiation width
ROC: Receiver operating characteristics
SIRS: Systemic inflammatory response syndrome
SPACE: Sepsis in the acutely ill patient in the emergency room
UMCU: University Medical Centre Utrecht
